# Effectiveness of postoperative elemental diet (Elental®) in elderly patients after gastrectomy

**DOI:** 10.1186/s12957-016-1013-3

**Published:** 2016-10-19

**Authors:** Yu Ohkura, Shusuke Haruta, Tsuyoshi Tanaka, Masaki Ueno, Harushi Udagawa

**Affiliations:** Hepato Pancreato Biliary Surgery Unit, Department of Gastroenterological Surgery, Toranomon Hospital, 2-2-2 Toranomon, Minato-ku, Tokyo 105-8470 Japan

**Keywords:** Elemental diet, Elderly patients, Gastrectomy

## Abstract

**Background:**

We aimed to investigate the efficacy of postoperative early intervention with an elemental diet to reduce weight loss and enhance recovery after gastrectomy. Nutritional status and gastrointestinal immune function tend to worsen, and postoperative weight loss is inevitable in these patients; therefore, improvement in their postoperative condition is important, especially in gastric cancer patients aged ≥80 years.

**Methods:**

Clinical outcomes and postoperative nutritional status were compared between 21 and 22 consecutive elderly patients aged ≥80 years who underwent distal gastrectomy before and after the introduction of postoperative oral elemental diet (Elental®, 300 kcal/day), respectively, between October 2011 and June 2016.

**Results:**

A significant reduction in postoperative complications was noted in the nutrition support group (N-group) as compared with the control group (C-group). In particular, the prevalence of systemic complications was significantly lower in the N-group (33.3 vs. 4.5 %, *p* = 0.015), whereas no significant difference was observed in the prevalence of locoregional complications. The percentage of weight loss and reduction in BMI from 1 month to 1 year after surgery was significantly lower in the N-group (*p* = 0.012 each). The nutrition status (albumin, total protein, hemoglobin, and C-reactive protein levels) at 1 month after surgery showed improvements (*p* = 0.005, *p* = 0.048), and hospital stay was decreased in the N-group as compared to the C-group (16.0 vs. 12.5 days, *p* = 0.041).

**Conclusions:**

Early intervention with an elemental diet after distal gastrectomy is valuable for reducing perioperative weight loss and improving nutritional management and may be associated with enhanced postoperative recovery in elderly patients.

## Background

Gastric cancer is a common cancer worldwide and has the fifth highest prevalence rate [1]. The prevalence rate continues to increase, particularly in East Asia [2]. Further, with population aging in recent years, the proportion of elderly patients with gastric cancer undergoing gastrectomy has also increased [3, 4]. Nutritional status and gastrointestinal immune function of gastric cancer patients tend to worsen postoperatively. Moreover, postoperative weight loss is inevitable, and improving the postoperative condition of these patients has therefore become an important concern in recent years [5–9]. Postoperative weight loss in elderly patients is of particular concern, as it may reduce their quality of life (QOL), increase susceptibility to complications (e.g., postoperative pneumonia and disuse syndrome), extend hospital stay, and decrease the survival rate. Growing attention is being paid to perioperative management using the enhanced recovery after surgery (ERAS) protocol, which aims to reduce surgical invasiveness and postoperative complications and shorten hospital stay. Several studies have reported the efficacy of early postoperative intervention with an oral elemental diet [10–12]. However, the benefits of early intervention including that on weight loss in elderly patients have not yet been shown. In this study, we introduced early intervention with an elemental diet after gastric cancer surgery in May 2014, with the aim of reducing postoperative weight loss and prevalence of postoperative complications; further, we evaluated the effects of this intervention on systemic nutritional status and early postoperative recovery in patients aged ≥80 years.

## Methods

### Study population

The initial cohort included 459 patients who underwent distal gastrectomy for gastric cancer in the Department of Gastroenterological Surgery, Toranomon Hospital, between October 2011 and June 2016. Among them, 48 patients aged ≥80 years were selected for inclusion in this study. Overall, 22 and 26 patients underwent gastrectomy before and after May 2014, respectively, the time point at which the early postoperative intervention of an oral elemental diet (Elental®: Ajinomoto Pharma Co., Tokyo, Japan) was introduced to promote early recovery and improvement of nutritional status after surgery. Elental® (300 kcal/day) was given from postoperative day 2 (post-gastrectomy) for 4–6 weeks. In this study, the primary endpoint was the effect of the oral elemental diet with respect to short-term outcomes and postoperative nutritional status in patients aged ≥80 years. Short-term surgical outcomes were surgical complications and the duration of hospital stay, and the nutritional status was assessed based on the percentage of weight loss and changes in hemoglobin (Hb), albumin, total protein (TP), and C-reactive protein (CRP) levels as compared to postoperative levels at 7 days after surgery.

The preoperative body weight was defined as the body weight measured on the day of surgery. The body weight was then measured again at 1 month after surgery (postoperative weight) to calculate the percentage of postoperative weight loss (= [postoperative weight − preoperative weight]/preoperative weight × 100). The body mass index (BMI) was then measured again at 1 month after surgery to calculate the rate of reduction of postoperative BMI (= [postoperative BMI − preoperative BMI]/preoperative BMI × 100). Blood levels of hemoglobin (Hb), albumin, total protein (TP), and C-reactive protein (CRP) were measured on postoperative day (POD) 7 and at 1 month after surgery to obtain the improvement rate (= [postoperative levels at 1 month after surgery − postoperative levels at 7 days after surgery]/postoperative levels at 7 days after surgery × 100). Surgical complications ≥grade 2 according to the Clavien-Dindo classification [13] were further grouped into systemic and locoregional complications in this study. Tumor staging was based on the UICC TNM classification version 7 [14]. This study was approved by the Institutional Review Board of Toranomon Hospital, Japan.

### Surgery

In accordance with the Japanese Gastric Cancer Treatment Guidelines [15], laparoscopic surgery was performed in patients with cStage I cancer while open surgery was performed in those with cStage II. D1+ or D2 lymph node dissection was performed depending on cancer progress and surgical risks. Gastric reconstruction was performed with the Roux-en-Y anastomosis.

### Statistical analysis

Pairwise differences in proportions and medians were analyzed by the chi-square test, Fisher’s exact test, or Mann-Whitney *U* test, as appropriate. All statistical analyses were performed using the Statistical Package for the Social Sciences (SPSS) version 19.0J for Windows (SPSS Inc., Chicago, IL). For all analyses, differences were considered statistically significant at *p* < 0.05.

## Results

### Patient characteristics

**Fig. 1:**
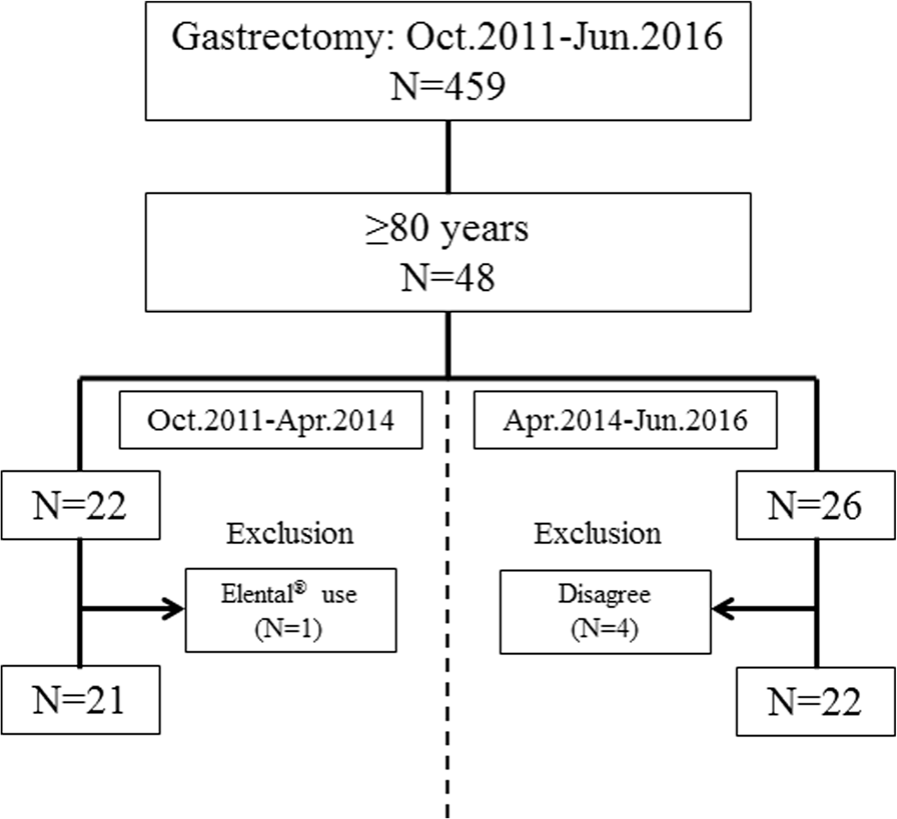
Study flow diagram. Our hospital introduced early intervention with an oral elemental diet from May 2014. Subjects were divided into two groups as follows: those who had not received the intervention (C-group) and those who received the intervention diet (N-group)

Patients who had not received the elemental diet formed the control group (C-group) while those who were given the elemental diet in the early postoperative period were included in the nutrition support group (N-group). One patient who was receiving an oral elemental diet and four patients who did not wish to receive nutritional support therapy were excluded from the C-group and N-group, respectively. Thus, the number of patients was 21 in the C-group and 22 in the N-group. The effect of early introduction of Elental® after surgery was compared between the two groups (Fig. 1).

The characteristics of the patients aged ≥80 years in the C- and N-groups (total, 43 patients) are summarized in Table 1. There were no differences in age, sex, ASA (American Society of Anesthesiologists) score, BMI, operative approach, clinical stage, operative duration, blood loss, or adjuvant chemotherapy between the two groups.

**Table 1:** Clinicopathological characteristics of the 43 elderly patients

	C-group (*n* = 21)	N-group (*n* = 22)	*p* value
Age: median (range) (years)	82.7 (80–89)	82.8 (80–91)	0.548
Sex			0.907
Male	13	14	
Female	8	8	
ASA			0.595
1–2	18	20	
3	3	2	
BMI	21.5	22.2	0.528
Operative approach			0.477
Open	14	12	
Laparoscopic	7	10	
cStage			0.323
I	14	12	
II	3	5	
III	4	4	
IV	0	1	
Operative duration (min)	242 (195–528)	278 (200–417)	0.215
Blood loss (mL)	200 (0–963)	248 (0–1667)	0.827
Adjuvant chemotherapy	3 (14.3 %)	2 (9.1 %)	0.595

### Postoperative short-term outcomes

Short-term outcomes and the results of nutritional status assessments in the two groups are shown in Table 2. Surgical complications were found in eight patients (nine complications) in the C-group and in two patients (three complications) in the N-group. The overall morbidity rate tended to be decreased in the N-group (9.1 %) than in the C-group (38.1 %) (*p* = 0.024). There were no deaths in either group. When the prevalence rates of systemic complications (delirium, pneumonia, pleural effusion, and cholecystitis) and locoregional complications (delayed gastric emptying, ileus, and abdominal abscess) were separately compared between the two groups, the prevalence of systemic complications was significantly lower in the N-group (4.5 %) than in the C-group (33.3 %) (*p* = 0.015). The median length of the postoperative hospital stay was significantly shorter in the N-group (12.5 days) than in the C-group (16.0 days) (*p* = 0.041).

**Table 2:** Postoperative short-term outcomes and nutritional status

	C-group (n = 21)	N-group (n = 22)	p value
Morbidity	8 (38.1 %)	2 (9.1 %)	0.024
Systemic complications	7 (33.3 %)	1 (4.5 %)	0.015
Delirium	3	0	
Pneumonia	3	1	
Pleural effusion	1	0	
Cholecystitis	1	0	
Locoregional complications	2 (9.5 %)	2 (9.1 %)	0.961
Delayed gastric emptying	1	0	
Ileus	1	1	
Abdominal abscess	0	1	
Mortality	0	0	
Postoperative hospital days (range)	16.0 (10–40)	12.5 (8–24)	0.041
Dietary intake calories (kcal/day) (range)	910 (560–1600)	980 (560–1570)	0.668
Weight loss rate at 1 month after gastrectomy (%)	8.43	5.38	0.012
Rate of reduction of BMI at 1 month after gastrectomy (%)	8.76	4.78	0.012

### Nutrition support outcomes

**Fig. 2:**
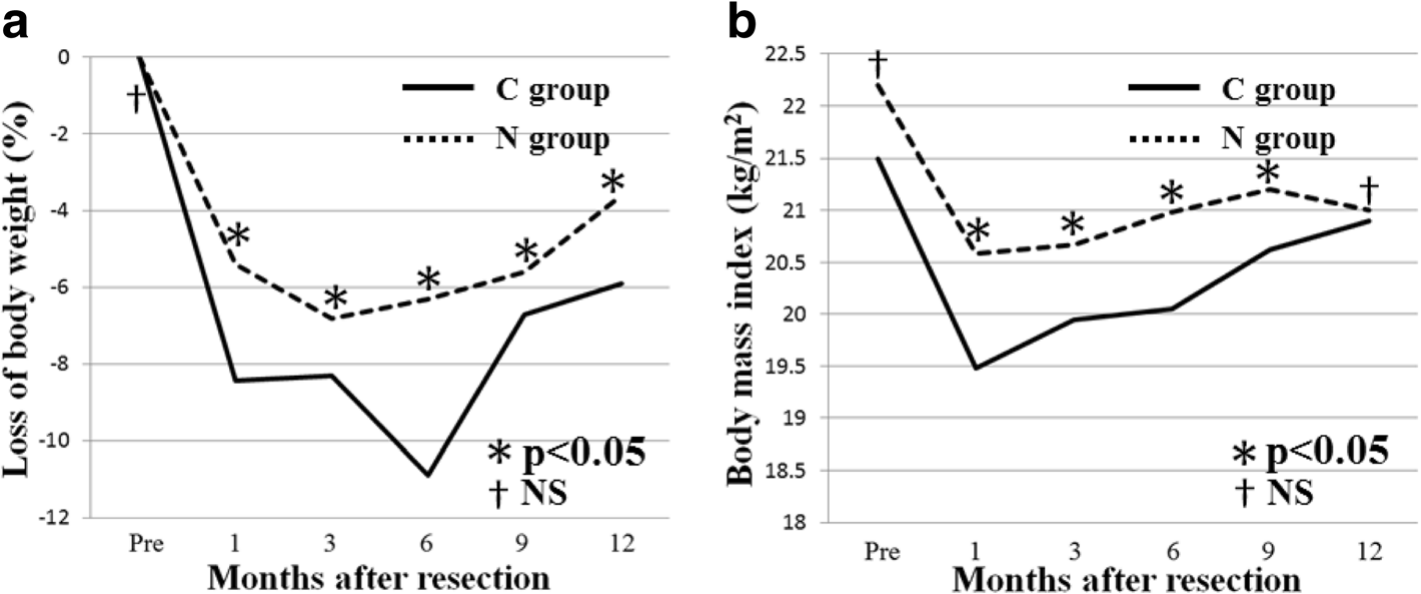
Trends in long-term outcomes up to 12 months after surgery. **a** Trends in loss of body weight (*p* = 0.012). **b** Trends in BMI (*p* = 0.012)

**Fig. 3:**
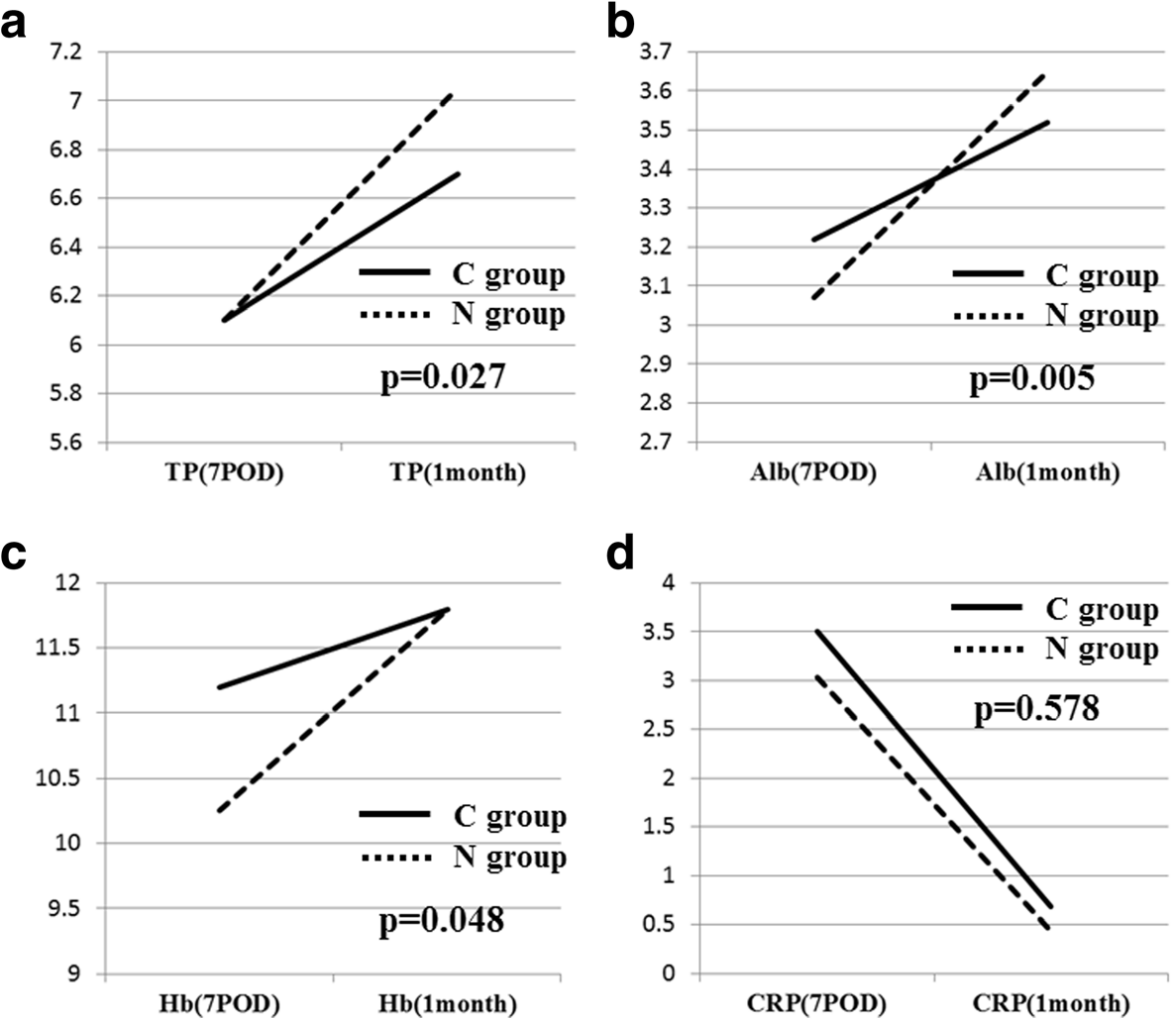
Improvement rates in total protein, albumin, and Hb levels at 1 month after surgery. The rates were significantly higher in the N-group than in the C-group. **a** C-group vs. N-group, 12.9 vs. 9.1 % for total protein levels (*p* = 0.027). **b** C-group vs. N-group, 9.3 vs. 18.9 % for albumin levels (*p* = 0.005). **c** C-group vs. N-group, 5.3 vs. 15.1 % for Hb level (*p* = 0.048). **d** CRP levels were not significantly different (*p* = 0.578)

First, no significant difference was observed in the median dietary intake of calories after surgery (C, 910 kcal/day; N, 980 kcal/day) (*p* = 0.668). The percentage of weight loss at 1 month after surgery was −8.43 % in the C-group and −5.38 % in the N-group; the difference between the two groups was significant (*p* = 0.012). The rate of reduction of BMI at 1 month after surgery was −8.76 % in the C-group and −4.78 % in the N-group, a significant difference between the groups (*p* = 0.012) (Table 2). Figure 2 shows the trends in these two variables (loss of body weight and BMI) up to 12 months after surgery as long-term outcomes. A significant reduction of weight loss and a smaller decrease in BMI in the N-group was noted in the period from preoperative assessment up to 12 months postoperatively. We also measured blood levels of TP, albumin, Hb, and CRP as indicators of nutritional status. The improvement rates in TP, albumin, and Hb levels at 1 month after surgery were significantly higher in the N-group than in the C-group: 12.9 vs. 9.1 % (*p* = 0.027) for TP levels, 18.9 vs. 9.3 % (*p* = 0.005) for albumin levels, and 15.1 vs. 5.3 % (*p* = 0.048) for Hb levels (Fig. 3a–c). On the other hand, CRP levels showed no significant difference (Fig. 3d).

## Discussion

The elemental diet used in the present study (Elental®) is low in fat, is a good source of nitrogen and amino acids, and rarely requires a fully functional digestive system. Thus, it is an easily absorbable, calorically dense agent suitable for enteral feeding. Further, the gastric emptying time for lipid soup has been reported to be significantly longer than that for non-lipid soup [16]; therefore, we considered that the low-lipid Elental® was less likely to lead to delayed gastric emptying. Because it only mildly irritates the gastrointestinal tract, this diet has been used for the treatment and perioperative nutritional management of patients with inflammatory bowel disease such as Crohn’s disease [17–19]. Therefore, a low burden is expected following the administration of this diet in the postoperative gastrointestinal tract with impaired function. Previous reports have demonstrated that early postoperative feeding is of value in patients undergoing a major operation of the upper gastrointestinal tract because early postoperative jejunal feeding of an elemental diet supplies higher amounts of nutrients and results in decreased weight loss [20–23]. It was also reported that Elental® does not seriously affect blood sugar control in patients with diabetes [24, 25], suggesting that its interference in perioperative blood sugar control is of minimal concern. In good agreement with these previous studies, we found no effect of Elental® on perioperative blood sugar control (data not shown). At Toranomon Hospital, we introduced early postoperative intervention with an oral elemental diet in May 2014, with the aim of reducing weight loss and prevalence of postoperative complications; accordingly, we evaluated its effects on systemic nutritional status and early postoperative recovery in patients aged ≥80 years.

The overall proportion of surgical complications ≥grade 2 was significantly lower in the N-group than in the C-group (*p* = 0.024). Elderly individuals are likely to have limited reserve capacity because of functional impairment of organs and existence of multiple underlying conditions. Decreases in activities of daily living (ADL) after surgery are prominent in elderly patients; therefore, they are expected to have higher rates of systemic complications as compared to young patients. Our findings showed that the proportion of systemic complications was significantly lower in the N-group than in the C-group (*p* = 0.015). Early intervention with an oral nutrient is likely to promote early mobilization and improvement of ADL. Thus, it may reduce the risk of postoperative delirium and contribute to the prevention of postoperative pneumonia. In other words, early postoperative intervention with an oral nutrient may reduce systemic complications that are particularly common in elderly patients. Moreover, the significant decrease in the prevalence of systemic complications ≥grade 2 according to the Clavien-Dindo classification in the N-group as compared with the C-group was likely to have contributed to the significantly shorter postoperative hospital stay noted in the N-group (12.5 days) than that in the C-group (16.0 days) (*p* = 0.041).

In this study, nutritional status was assessed in three ways, namely, percentage of weight loss, reduction in BMI, and the improvement rate in Hb and albumin levels after surgery as compared with preoperative levels. Postoperative weight loss is almost inevitable due to the decrease in food intake after surgery for gastric cancer. Further, poor nutrition postoperatively is a risk factor for ADL deterioration in elderly patients. It is generally said that poor nutrition after surgery has the following negative effects: delayed wound healing, increased postoperative complications, impaired immunity (development of infections), prolonged postoperative hospital stay, increased mortality, decreased muscle mass, decreased protein levels in internal organs, exacerbation of pressure ulcers, and deterioration of ADL and QOL [26, 27]. In this study, early oral administration of nutrients significantly reduced the percentage of weight loss and reduction in BMI at 1 month after surgery from −8.43 to −5.38 % (*p* = 0.012) and from −8.76 to −4.78 % (*p* = 0.012), respectively. With respect to long-term outcomes, the N-group showed faster prevention of weight loss and significantly greater increases in weight and BMI between 1–3 months and 12 months postoperatively. Moreover, this intervention resulted in significant improvements in TP (*p* = 0.027), albumin (*p* = 0.005), and Hb (*p* = 0.048) levels. Taken together, the reduction in weight loss and the smaller decrease in BMI as well as the increases in TP, albumin, and Hb levels after the surgical treatment of gastric cancer indicate that the early administration of an elemental diet improves nutritional status.

Further studies are necessary to confirm how the improvement of nutritional status in the early postoperative stage affects QOL and long-term prognosis.

Our study has certain limitations, including its retrospective nature and small sample size. However, the perioperative management in our study population was similar to the current data from a prospectively collected database for consecutive patients. In addition, the significant reduction in weight loss and prevalence of postoperative complications as well as the improved systemic nutritional status appear to be strongly associated with enhanced recovery and decreased duration of hospital stay after gastrectomy in elderly patients. An external validation study that includes a larger number of patients would be needed to confirm the current observations.

## Conclusions

Early intervention with the elemental diet reduced weight loss and the decrease in BMI and improved blood TP, albumin, and Hb levels over the early and long-term postoperative stages. Such an elemental diet may be a feasible nutrition management option in the ERAS protocol after gastrectomy in patients aged ≥80 years.

## Abbreviations

ADL: Activities of daily living
ASA: American Society of Anesthesiologists
BMI: Body mass index
POD: Postoperative day
QOL: Quality of life
